# Characterization of *RUNX1T1*, an Adipogenesis Regulator in Ovine Preadipocyte Differentiation

**DOI:** 10.3390/ijms19051300

**Published:** 2018-04-26

**Authors:** Kaiping Deng, Caifang Ren, Zifei Liu, Xiaoxiao Gao, Yixuan Fan, Guomin Zhang, Yanli Zhang, Ei-Samahy MA, Feng Wang, Peihua You

**Affiliations:** 1 Institute of Sheep and Goat Science; Nanjing Agricultural University, Nanjing 210095, China; 2017205013@njau.edu.cn (K.D.); 2014205007@njau.edu.cn (C.R.); 2016105022@njau.edu.cn (Z.L.); 2017205002@njau.edu.cn (X.G.); fanyixuan@njau.edu.cn (Y.F.); zhangguomin@njau.edu.cn (G.Z.); zhangyanli@njau.edu.cn (Y.Z.); 2016105031@njau.edu.cn (E.-S.M.); 2 Portal Agri-Industries Co., Ltd., Xingdian Street, Pikou District, Nanjing 210095, China; ph-u@163.com

**Keywords:** *RUNX1T1*, subcutaneous fat, fat development, adipogenesis

## Abstract

Runt-related transcription factor 1 translocation partner 1 (RUNX1T1), a potential novel regulator of adipogenesis, exists in two splice variants: a long (RUNX1T1-L) and a short (RUNX1T1-S) isoform. However, there is no data showing the existence of *RUNX1T1* in ovine subcutaneous fat at different stages of developmental and its role on ovine adipogenesis. Therefore, the objectives of this study were to evaluate the presence of *RUNX1T1* in subcutaneous fat of five-day-old to 24-month-old sheep and to investigate the role of *RUNX1T1* in ovine adipogenesis. In this study, we detected a 1829 bp cDNA fragment of *RUNX1T1* which contains a 1815 bp coding sequence that encodes 602-amino acid and 14 bp of 5′ untranslated region, respectively. The amino acid sequence of RUNX1T1 has 31.18–94.21% homology with other species’ protein sequences. During fat development, the RUNX1T1 protein expression was higher in subcutaneous fat of 24-month-old Hu sheep. In addition, the expression of *RUNX1T1-L* mRNA decreased first, then subsequently increased during ovine preadipocyte differentiation. Knockdown of *RUNX1T1-L* in ovine preadipocytes promoted preadipocyte differentiation and lipid accumulation. Taken together, our data suggests that *RUNX1T1* is an important functional molecule in adipogenesis. Moreover, it showed for the first time that *RUNX1T1-L* was negatively correlated with the ovine preadipocyte differentiation.

## 1. Introduction

Adipocyte differentiation is a complex process that involves two stages; (i) commitment of mesenchymal precursors to the fate of preadipocyte and (ii) terminal differentiation [1]. Under appropriate stimulation, preadipocytes undergo cellular morphology changes and eventually differentiate into round mature adipocytes [2]. Adipocyte differentiation involves an interaction of peroxisome proliferator-activated receptor gamma (PPARγ) with CCAAT/enhancer-binding protein (C/EBP) transcription factors [3,4]. Recent evidence indicated that Runt-related transcription factor 1 translocation partner 1 (RUNX1T1) acts as a potential novel regulator factor of adipogenesis and is actively involved in the mitotic clonal expansion phases of adipogenesis [5,6].

RUNX1T1 is a member of the runt-related transcription factor (RUNX) family of transcription factor, also known as eight twenty one (ETO) and myeloid translocation gene 8 (MTG8), which is involved in the regulation of proliferation and differentiation of hematopoietic stem cells [7,8]. Several studies have shown that the *RUNX1T1* gene is widely expressed in many human tissues, with the highest expression levels in brain and heart [9,10]. Current research on *RUNX1T1* mainly focuses on leukemia, cancer and neuronal differentiation [11,12,13], however, there are few reports about adipogenesis. In 3T3-L1 cells, *RUNX1T1* exists in two splice variants; a long and a short isoform. In addition, the expression of *RUNX1T1* isoforms is regulated by the gene fat mass and obesity-associated (*FTO*) [14]. Previous studies in mouse primary adipocytes have found that overexpression of the long isoform of *RUNX1T1* (*RUNX1T1-L*) inhibits adipocyte differentiation [5], conversely, increased expression of the short isoform of *RUNX1T1* (*RUNX1T1-S*) stimulates adipogenesis [6]. These reports further demonstrate the potential role of *RUNX1T1* in regulating adipogenesis.

Notably, recent studies in 3T3-L1 cells and mice showed that FTO plays a role in early adipogenesis to increase the number of adipocytes by regulating alternative splicing of *RUNX1T1* mRNA [6,14]. However, the mechanism by which *RUNX1T1* affects adipogenesis has been elusive. In addition, little is known about the function of *RUNX1T1* in fat development in large mammalian species. Therefore, we explored whether *RUNX1T1* modulates preadipocyte differentiation derived from native cells of sheep. Although several evidences [9,10] have demonstrated the presence of *RUNX1T1* in human and mouse tissues, there is no data available for ovine adipose tissue. Therefore, in the present study, we cloned the ovine *RUNX1T1* coding sequence and further investigated whether *RUNX1T1* exists in the adipose tissue of sheep. 

## 2. Results

### 2.1. cDNA Cloning and Sequence Analysis

**Figure 1:**
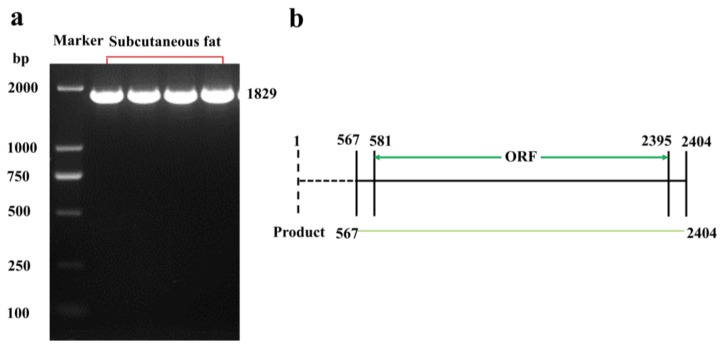
RUNX1T1-coding sequence (RUNX1T1-CDS) primers were designed for amplifying the open reading frame (ORF) of *RUNX1T1* (**a**) and the sketch of *RUNX1T* amplification product from Hu lamb (**b**). The four lanes in the red bracket represented *RUNX1T1* amplification product using subcutaneous fat cDNA (**a**). The dashed line represents the 5′ untranslated region of *RUNX1T1* (**b**)*.*

**Figure 2:**
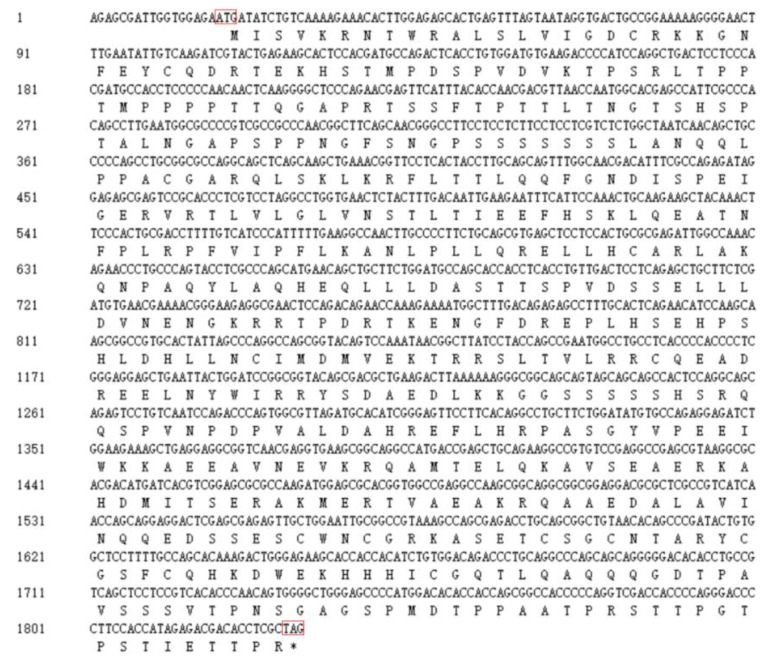
The cDNA fragment and predicted protein sequences of ovine RUNX1T1. The start and stop codons are framed in red. The protein sequence was predicted by the BLAST tool of DNAMAN (version 6.0, LynnonBiosoft, San Ramon, CA, USA) and is presented under the coding sequence. All sequences were aligned by the DNAMAN (version 6.0, LynnonBiosoft) software. * means the translation is terminated.

**Figure 3:**
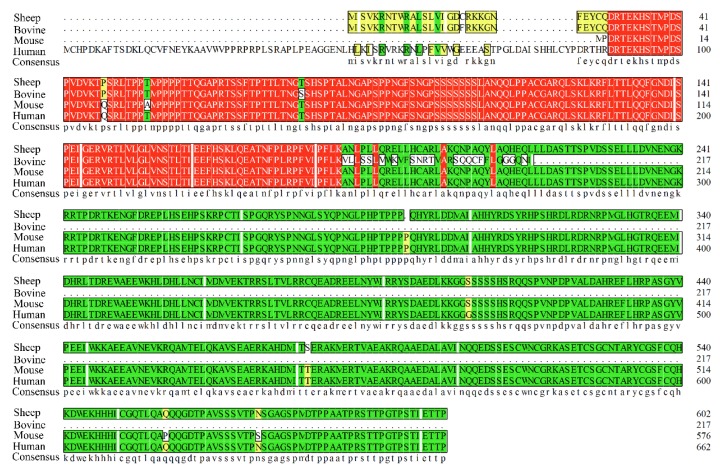
Comparison of the bovine, mouse, and human RUNX1T1 amino acid sequences. The RUNX1T1 amino acid sequence (Accession No.: MH063277) was aligned to those of bovine (Accession No.: NP_001092855), murine (Accession No.: EDL05616), and human (Accession No.: NP_001185608.1) by DNAMAN. The same amino acid residues in four, three and two species are highlighted in red, green, yellow respectively, while the amino acid residues present in only one species are highlighted in white. The apostrophes indicates that these amino acid sequences are absent.

To obtain the *RUNX1T1* cDNA fragment (1829 bp) of sheep, we designed a pair of PCR primers (Table S1) and used these to undertake reverse transcription of the mRNA from subcutaneous fat of Hu sheep (Figure 1), then submitted the obtained cDNA fragment to the GenBank (https://www.ncbi.nlm.nih.gov/genbank/; accession number MH063277). The sequence analysis indicated that the coding sequence of *RUNX1T1* was 1815 bp and encoded a 602-amino acid protein with a predicted molecular weight of 67 kDa (Figure 2). Comparison of the predicted protein with the homologous proteins from other species—including bovine, mouse, and human—using DNAMAN (version 6.0, LynnonBiosoft, San Ramon, CA, USA) showed high homology with mouse (94.21%) and human (86.73%) RUNX1T1 and low sequence identity with bovine (31.18%) RUNX1T1 (Figure 3).

### 2.2. Expression Pattern of RUNX1T1 Isoforms in Different Tissues of Hu Sheep

**Figure 4:**
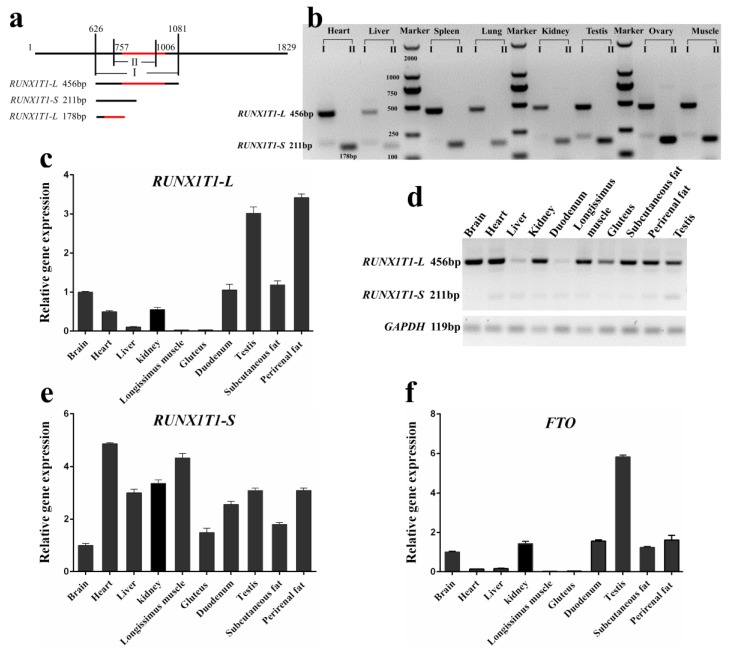
Expression pattern of *RUNX1T1* isoforms in different tissues from 6-month-old Hu sheep. (**a**) Primer I was designed for identifying *RUNX1T1* isoforms, primer II was used for qPCR of *RUNX1T1-L* mRNA. The red line represents the region which is present in the long isoform and absent in the short isoform. Depending on isoforms, bands of either 456 bp or 211 bp can be detected (**b**). Real-time PCR of mRNA for *RUNX1T1-L* (178 bp; (**c**)) and *FTO* (**f**) in different tissues. Expression of the *RUNX1T1-S* (211 bp) in different tissues were determined by semi-qRT-PCR (**d**,**e**). Expression of gene was normalized to that of glyceraldehyde-3-phosphate dehydrogenase (*GAPDH*), and relative to the expression in brain. Quantitative data are represented as the mean ± SEM (*n* = 5).

*RUNX1T1* exists in two splice variants—a long and a short isoform—in 3T3-L1 cells [14]. These isoforms were also found in different tissues of Hu sheep (Figure 4b). The PCR product of the constitutive *RUNX1T1-L* is 456 bp, while *RUNX1T1-S*, skipping the sequence of 757 to 1006, is 211 bp (Figure 4a). The PCR products were verified by sequencing analysis. To evaluate the expression pattern of *RUNX1T1* isoforms, we first analyzed mRNA levels of the *RUNX1T1-L* and *RUNX1T1-S* in various Hu sheep tissues. It was found that *RUNX1T1-L* was expressed at much higher levels than *RUNX1T1-S* in these tissues (Figure 4b; *p* < 0.5). In addition, the mRNA levels of *RUNX1T1-L* in testis and adipose tissue were higher than that in other tissues (Figure 4c; *p* < 0.5). However, the expression of *RUNX1T1-S* in adipose tissue was similar to that in liver, kidney, testis, and duodenum (Figure 4d,e; *p* > 0.5). It has been suggested that *FTO* can affect the expression pattern of short and long isoforms of *RUNX1T1* by regulating the *RUNX1T1* mRNA splicing [14]. We also found that the expression trend of *FTO* in different tissues is similar to that of *RUNX1T1-L* (Figure 4f).

### 2.3. Expression Pattern of RUNX1T1 Isoforms and Adipogenic Marker Genes in Subcutaneous Fat Tissue at Different Developmental Stages

**Figure 5:**
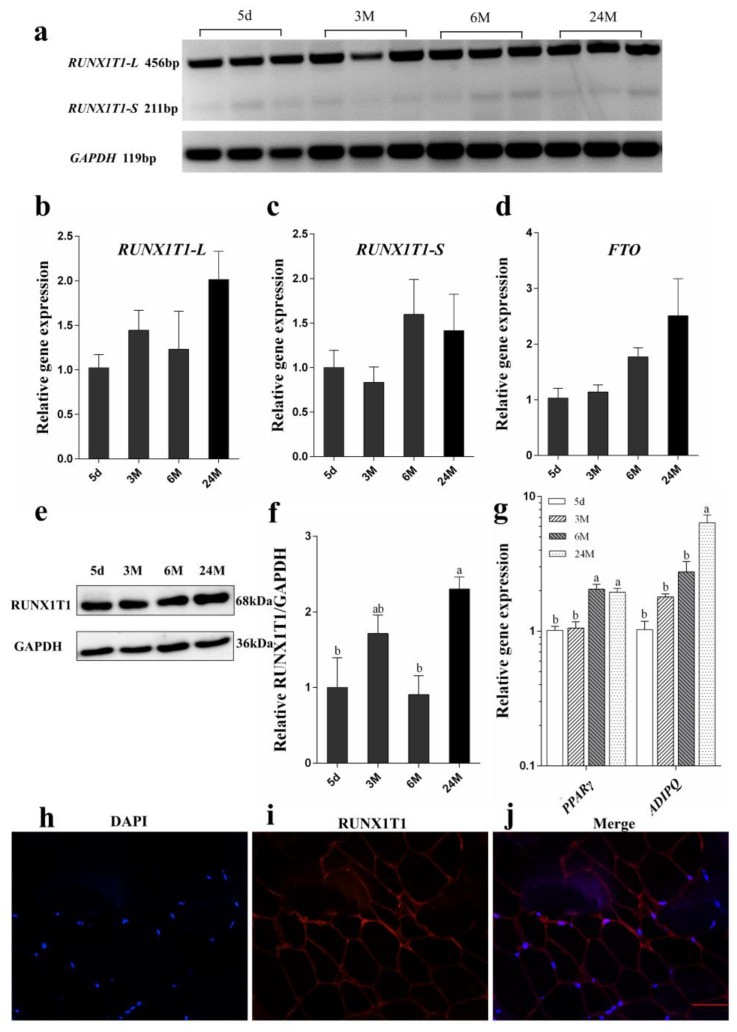
Expression patterns of *RUNX1T1* isoforms in the subcutaneous fat. The temporal expression of *RUNX1T1* isoforms (**a**–**c**) and *FTO* mRNA (**d**) and RUNX1T1 protein (**e**) in the subcutaneous fat of Hu sheep at different ages were measured using Real-time PCR and Western blot (5 d, 3 M, 6 M and 24 M). d means day, M means month. Semi-quantitative analysis of the relative RUNX1T1 protein expression levels in the subcutaneous fat at different developmental stages was undertaken (**f**). Real-time PCR was performed on different developmental stages of subcutaneous fat to determine the expression level of marker genes (**g**), and immunofluorescence localization of RUNX1T1 in the subcutaneous fat of 24-month-old Hu sheep (**h**–**j**). Blue color indicates 4′,6-diamidino-2-phenylindole (DAPI) staining of the nuclei (**h**). Red color indicates the expression of RUNX1T1 (**i**). Pictures in (**g**) and (**h**) were merged (**j**). Scale bars = 100 µm. One-way ANOVA, and bars with different letters are significantly different (*p* < 0.05). Quantitative data are shown as mean ± SEM (*n* = 5).

Results of Real-time PCR showed that the mRNA levels of adipogenic marker genes, *PPARγ* and adiponectin (*ADIPOQ*) were increased during development stages (Figure 5g), which indicates cells were undergoing differentiation. It has been suggested that *RUNX1T1* plays an important role in regulating adipogenesis [6]. The temporal expression of *RUNX1T1* mRNA and protein in subcutaneous fat tissue at different developmental stages was detected by Real-time PCR (Figure 5a–c) and Western blot analysis (Figure 5e,f), respectively. The long and short isoform of *RUNX1T1* in subcutaneous fat showed similar expression pattern amongst different age groups (*p* > 0.5). Likewise, the expression of *FTO* in different age groups were similar (Figure 5d). However, the RUNX1T1 protein in subcutaneous fat of 24-month-old Hu sheep was higher than other developmental stages (*p* < 0.05). The original western blots image of RUNX1T1 and GAPDH were shown in Figure S1. In addition, we found that RUNX1T1 protein was located in the cytoplasm of subcutaneous fat by immunofluorescence (Figure 5h–j).

### 2.4. Expression Pattern of RUNX1T1 Isoforms during Ovine Preadipocyte Differentiation

**Figure 6:**
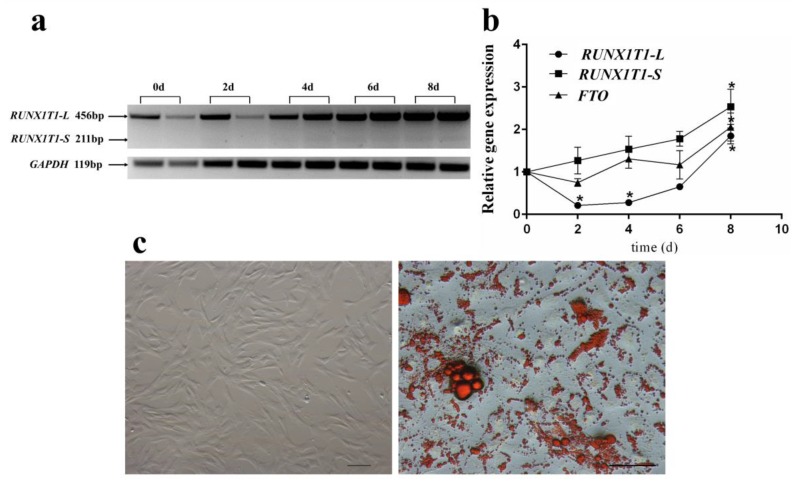
Expression patterns of *RUNX1T1* isoforms during ovine preadipocyte differentiation. The temporal expression pattern of *RUNX1T1* isoforms and *FTO* during ovine preadipocyte differentiation (**a**,**b**); image of newly isolated and adipogenic differentiated ovine preadipocytes (**c**). Scale bars = 100 µm. One-way ANOVA followed by comparison of all time points to day 0 with Tuckey *post hoc* analysis, * *p* < 0.05. Quantitative data are represented as the mean ± SEM (*n* = 4).

To obtain an ovine adipogenesis model, we successfully isolated ovine primary adipocytes (Figure 6c). Based on an in vitro ovine adipogenesis model, we observed that the expression of *RUNX1T1-S* and *FTO* were increasing during day 0 to 8 of adipogenic differentiation (Figure 6a,b; *p* < 0.05). However, the *RUNX1T1-L* expression was decreased first and then increased during preadipocyte differentiation (*p* < 0.05).

### 2.5. RUNX1T1-L Knockdown Promoted Lipid Accumulation and Ovine Preadipocyte Differentiation

**Figure 7:**
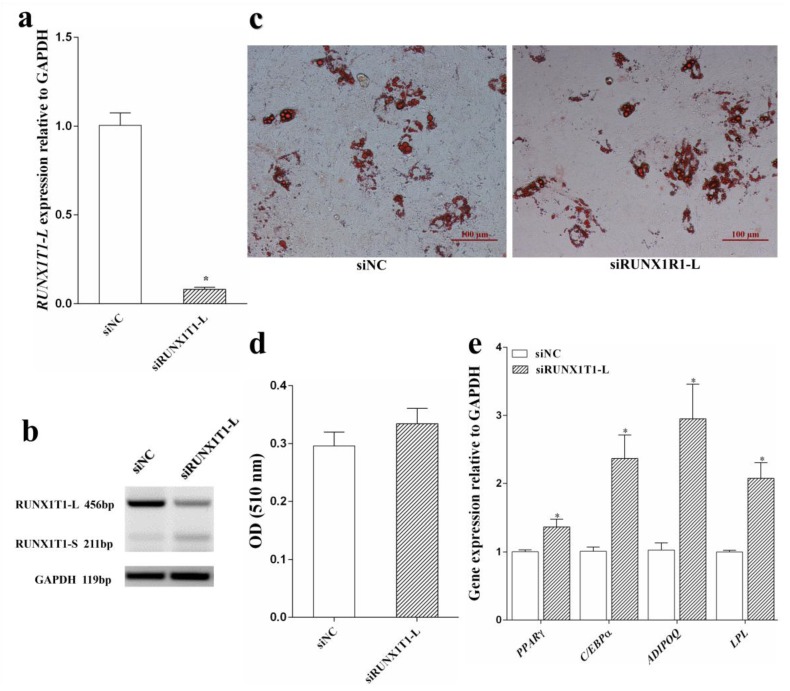
Adipogenic differentiation of ovine preadipocytes with *RUNX1T1-L* knockdown. siRUNX1T1-L and siNC were introduced into ovine preadipocytes when the density reached about 70%, and 48 h later, cells were subject to adipogenic differentiation (**a**). The expression of *RUNX1T1-L* was determined at 48 h post-transfection by Real-time PCR. Moreover, the expression of *RUNX1T1-S* was analyzed by semi-qRT-PCR (**b**). Ovine preadpocytes were transfected with siRUNX1T1-L or siNC and differentiated into mature adipocytes (**c**). Scale bars = 100 µm. After 8 days of differentiation, intracellular lipid droplets were stained using Oil Red-O. Lipid content was indirectly determined by measuring the optical density (OD) value at 510 nm on a spectrophotometer (**d**). Real-time PCR was performed on day 6 of adipogenic differentiation to determine the mRNA level of maker genes (**e**). Independent Students’ *t*-test. * *p* < 0.05 against siNC. Quantitative data are represented as the mean ± SEM (*n* = 4).

It has been suggested that overexpression of *RUNX1T1-L* attenuates lipid accumulation [14]. Based on previous findings, and the data of the *RUNX1T1-L* expression pattern in ovine preadipocyte and adipose tissue, we proposed that *RUNX1T1-L* may play a negative regulatory role in ovine preadipocyte differentiation. To verify this hypothesis, we performed *RUNX1T1-L* knockdown in ovine preadipocyte using siRUNX1T1-L. At 48 h after transfection, we found that siRUNX1T1-L transfection significantly decreased the expression of *RUNX1T1-L* in cells (Figure 7a; *p* < 0.05), however increased the expression of *RUNX1T1-S* (Figure 7b; *p* < 0.05). Oil Red-O staining and extraction assays showed that *RUNX1T1-L* knockdown promoted lipid accumulation in ovine adipocytes (Figure 7c,d). Furthermore, we performed Real-time PCR measurement and observed that on day 8 of differentiation, the mRNA levels of adipogenic marker genes, *PPARγ*, *C/EBPα*, *ADIPOQ* and lipoprotein lipase (*LPL*) were upregulated to different extents by *RUNX1T1-L* interference (Figure 7e; *p* < 0.05). 

## 3. Discussion

Sheep is an apropos animal model of adult metabolic disease research as well as the primary meat source of human diet in many countries [15,16]. However, studies on the function of *RUNX1T1* in ovine adipogenesis are rare. Although the two isoforms of *RUNX1T1* have distinctive expression patterns during preadipocyte differentiation, the potential function of this gene in fat development, such as ovine adipogenesis, has not received sufficient attention. In this study, the ovine *RUNX1T1* gene was cloned for the first time, and the coding sequence of this gene was obtained. In addition, we found that *RUNX1T1* can affect ovine preadipocyte differentiation.

In the present study, we cloned the *RUNX1T1* gene from the subcutaneous fat of Hu sheep. The results showed that the coding sequence of *RUNX1T1* was 1815 bp and encoded 602-amino acid. However, the *RUNX1T1* coding sequence encodes 271, 579, and 662 amino acids in cattle, mice, and humans, respectively [17,18]. The amino acid sequence of sheep RUNX1T1 is highly homologous to RUNX1T1 in mice (94.21%) and humans (86.73%), however has poor homology to RUNX1T1 in cattle (31.18%). As a member of the conserved RUNX transcription factor family, *RUNX1T1* exists in two splice variants; a long and a short isoform. Indeed, we found that the coding sequence of *RUNX1T1-L* is 245 bp longer than that of *RUNX1T1-S* [19]. In general, *RUNX1T1* is abundantly expressed in the heart and brain of human [20]. However, we found that *RUNX1T1-L* is abundant in sheep testis and adipose tissue in the present study. These discrepancies may be caused by species differences.

As a white fat tissue, subcutaneous fat is characterized by mature adipocytes containing large and unilocular lipid droplets. It is also an active endocrine organ involved in various activities, such as insulin sensitivity, glucose tolerance, lipid metabolism and deposition [21,22]. Moreover, previous studies have found that subcutaneous fat deposition is related to meat quality [23]. The growth and development of adipose tissue includes two important stages: (i) adipocyte proliferation and (ii) adipocyte hypertrophy [24]. Proliferation of adipocytes proceeds in late gestation, while adipocyte hypertrophy mainly occurs postnatally [25]. Previous studies have confirmed that *PPARγ* plays an essential role in maintaining mature adipocyte function [26,27]. In the present study, *PPARγ* and *ADIPOQ* were used as biomarkers of mature adipocytes, and their expression increased with increasing developmental stages, indicating more lipid droplet deposition in mature adipocytes. In addition, the expression level of RUNX1T1 protein in subcutaneous fat increased with age. These results indicate that *RUNX1T1* may play an important role in fat development in sheep.

Although there are few reports on the role of *RUNX1T1* in adipogenesis, *RUNX1T1* has been studied in-depth for its sophisticated functions since its initial identification [28,29]. In the formation of blood vessels, *RUNX1T1* was confirmed to serve as an important angiogenic driver for vasculogenesis [30]. However, previous studies reported that *RUNX1T1* has pleiotropic effects on fat development such as inhibiting CCAAT enhancer binding protein beta (*C/EBPβ*) activity during early adipogenesis and modulating preadipocyte differentiation through alternative splicing of *RUNX1T1* [5,31]. FTO is a nucleic acid demethylase that can demethylate *N*^6^-methyladenosine [32], thereby controlling alternative splicing of *RUNX1T1* mRNA [14]. Indeed, the expression of *RUNX1T1-S* increased during the differentiation of ovine preadipocytes, which was consistent with the expression of the *FTO* gene. These results indicated *RUNX1T1* may be a regulator of ovine preadipocyte differentiation.

The two isoforms of *RUNX1T1* are generally considered to have different functions in regulating adipogenesis [5,6]. Though previous studies did not distinguish differences between the long and short isoforms, overexpression of *RUNX1T1* was found to suppress adipogenesis by inhibiting the transcriptional cascade that results in adipocyte formation [5]. Considering that the expression level of the long isoform of *RUNX1T1* is higher than that of the short isoform in ovine adipose tissue and preadipocytes, the predominance of *RUNXT1-L* in regulating the process of adipogenesis is indicated.

To the best of our knowledge, this is the first study to assess the effect of the long isoform of *RUNX1T1* on ovine preadipocyte differentiation. In the present study, we observed that knockdown of *RUNX1T1-L* resulted in the loss of the long isoform, whereas the short isoform of *RUNX1T1* increased. The increase of short isoform may be due to the compensating effects of *RUNX1T1-L* interference [33]. In addition, knockdown of *RUNX1T1-L* promoted ovine preadipocyte differentiation and lipid accumulation. The predominant effect of *RUNX1T1-L* knockdown on preadipocyte differentiation may be related to the activation of *C/EBPβ* [5] and the increased expression of the short isoform [14]. RUNX1T1-L may bind to C/EBPβ, thereby blocking its DNA-binding activity and transcription of its downstream genes (such as *PPARγ*) [34], inhibiting preadipocyte differentiation. However, the mechanism by which the long isoform of *RUNX1T1* effects preadipocyte differentiation is unclear. Thus, further studies are still required to determine the role of the long isoform of *RUNX1T1* in ovine preadipocyte differentiation.

## 4. Material and Methods

All experimental design and procedures were performed in accordance with the approved Guidelines for Animal Experiments of Nanjing Agricultural University, China and were approved by the Animal Care and Use Committee of Nanjing Agricultural University, China (Approval ID: SYXK2011-0036; date: 6 December 2011).

### 4.1. Sample Collections

The experiment was carried out at the Jiangsu Taizhou Helen Sheep Industry Co., Ltd. A total of twenty Hu sheep were selected and divided into four groups according to the age. At the end of experiment, animals were slaughtered by exsanguination. At 30 min post-slaughter, brain, heart, liver, kidney, longissimus, gluteus, duodenum, testis, subcutaneous fat and perirenal fat were sampled. Samples were immediately frozen in liquid nitrogen for further study. One portion of sample was made into frozen sections for immunofluorescence; the other was stored at −80 °C for further analysis of gene and protein expression.

### 4.2. Primary Adipocyte Culture

Ovine primary adipocytes were isolated from the cervical subcutaneous fat tissue of 7-day-old Hu lambs under sterile conditions. The tissues were then minced and digested with collagenase (Sigma-Aldrich, St Louis, MO, USA) in Krebs-Ringer 2-[4-(2-hydroxyethyl)piperazin-1-yl]ethanesulfonic acid buffer (HEPES) for 1 h at 37 °C. The digested tissue was filtered with a 200 µm nylon mesh and then centrifuged at 1200 rpm for 5 min to obtain preadipocytes. The cell pellet was re-suspended and washed with phosphate buffered solution (PBS, Gibco, Grand Island, NY, USA) and finally cultured in Dulbecco modified eagle (DMEM, Gibco) containing 10% fetal bovine serum (Gibco) and 1% penicillin/streptomycin (Gibco). Preadipocyte differentiation was induced by supplementing the growth culture medium with 0.5 mM 3-isobutyl-1-methylxanthine (IBMX), 1µM dexamethasone, 20 µM rosiglitazone and 10 µg/mL insulin for 4 days. After this period, the culture medium was supplemented with 10 µg/mL insulin only. The whole adipogenic process of ovine preadipocytes took 8–10 days.

### 4.3. Small Interfering RNAs (siRNAs)

siRNAs targeting *RUNX1T1-L* and non-targeting control siRNA (NC siRNA) were purchased from Shanghai GenePharma (Shanghai, China). The siRNAs transfections were carried out using Lipofectamine 2000 reagent (Invitrogen, Carlsbad, CA, USA) according to the manufacturer’s protocol. Firstly, cells (2 × 10^5^) were seeded onto 6-well plates and incubated overnight. Then each well cell was transfected with 50 nM of *RUNX1T1-L* siRNA and NC siRNA, respectively. After transfection, cells were harvested at indicated time points. The *RUNX1T1-L* siRNA (siRUNX1T1-L) sequences were showed as follows: sense: 5′-GCUUUGACAGAGAGCCUUUTT-3′; antisense: 5′-AAAGGCUCUCUGUCAAAGCTT-3′, and the control siRNA (siNC) sequences are: sense: 5′-UUCUCCGAACGUGUCACGUTT-3′; antisense: 5′-ACGUGACACGUUCGGAGAATT-3′.

### 4.4. Oil Red-O Staining and Extraction Assay

Ovine adipocytes were washed with PBS and fixed in 10% formaldehyde formalin for 30 min at room temperature. After washing three times in PBS, cells were stained with a filtered 1% Oil Red-O (Sigma-Aldrich, St Louis, MO, USA) solution at room temperature for 30 min, washed again in PBS and visualized under an inverted microscope (Olympus, Tokyo, Japan). To quantify intracellular lipid content, the lipid droplets were extracted from Oil Red-O-stained adipocytes with pure isopropanol. The optical density of solution (OD value) was measured at 510 nm on a spectrophotometer (Thermo Fisher Scientific, Waltham, MA, USA).

### 4.5. RNA Isolation and cDNA Synthesis

Total RNA was extracted from different tissues and cells using Trizol reagent (Invitrogen, Carlsbad, CA, USA) according to the manufacturer’s protocol. The RNA quality (purity and integrity) was determined using ND-2000 spectrophotometer (NanoDrop Technologies, Wilmington, DE, USA) and 1% agarose gel electrophoresis. Thereafter, cDNA was synthesized using reverse transcription reagent kits (Takara, Dalian, China).

### 4.6. Cloning of RUNX1T1

To obtain the coding sequence of *RUNX1T1* in Hu Sheep, a pair of specific primers RUNX1T1-CDS were designed using Primer 5.0 software (PREMIER Biosoft, Palo Alto, CA, USA) (Table S1). The ovine subcutaneous fat cDNA was amplified by PCR. The PCR conditions were as follows; 94 °C for 5 min, 35 cycles of 98 °C for 10 s, 60 °C for 45 s, 72 °C for 45 s and 72 °C for 7 min. All PCR products were separated using 1.5% agarose gel. After purification, the target PCR products were cloned into a pClone007 Blunt Vector (TSINGKE Biological Technology, Beijing, China) and then transformed into *Escherichia coli* DH5a cells. Positive clones were randomly selected and sequenced at TSINGKE Biological Technology.

### 4.7. Real-time PCR Analysis for mRNA Expression

Real-time PCR was performed according to the protocol described in our previous study [35] for gene expression quantification. Gen Bank accession numbers and sequences of the corresponding Real-time PCR primer are listed in Table S1. At the same time, glyceraldehyde-3-phosphate dehydrogenase (*GAPDH*) was used to normalize the mRNA expression level. The relative gene expression of *RUNX1T1-L*, *FTO*, *PPARγ*, *C/EBPα*, *ADIPOQ*, and *LPL* were calculated with the comparative, efficiency-corrected 2^−ΔΔ*C*t^ method.

### 4.8. Semi-Quantitative Reverse Transcription Polymerase Chain Reaction (Semi-qRT-PCR)

The expression level of *RUNX1T1-S* was determined by semi qRT-PCR method. The PCR was carried out in 20 μL reaction volume containing 1 μL cDNA, 10 μL LA Taq PCR Master Mix (Takara, Dalian, China), 7.8 μL nuclease-free water and 0.6 μL each of forward and reverse primer pairs (10 pmol). To amplify ovine *GAPDH* (internal control) and *RUNX1T1-S*, a denaturing cycle of 10 min at 95 °C, followed by 30 cycles of (95 °C for 10 s, an annealing step at 60 °C for 30 s and extension step at 72 °C for 45 s), was performed for each PCR. The PCR products were separated by 1% agarose gel electrophoresis. Following, the images of the semi-qRT-PCR stained with ethidium bromide were analyzed using ImageJ 1.50i software (National Institutes of Health, Bethesda, MD, USA). The band intensity of the genes of interest was normalized to *GAPDH*.

### 4.9. Western Blot Analysis for Protein Expression

Protein samples were prepared using protein lysis buffer (Radio Immunoprecipitation Assay; Beyotime, Shanghai, China) with phenylmethanesulfonyl fluoride (PMSF; Beyotime). After incubation on ice for at least 20 min, the lysates were centrifuged to remove insoluble material, and protein concentrations were estimated by a BCA protein assay kit (Beyotime).

Approximately 20 µg of the protein lysates from each treatment group were run on 12% sodium dodecyl sulfate (SDS) polyacrylamide gel and transferred to polyvinylidene fluoride membranes (Millipore; Billerica, MA, USA). Membranes were blocked for 1 h at room temperature in 5% (*w*/*v*) fat-free milk, and then incubated overnight at 4 °C with corresponding primary antibodies to RUNX1T1 (1:1000, Proteintech, Chicago, IL, USA) and GAPDH (1:8000, Proteintech) as an internal control. After washing with Tris buffered saline with Tween (TBST), membranes were incubated with the appropriate secondary antibody (1:1000, horseradish peroxidase (HRP)-labeled goat anti-rabbit IgG for RUNX1T1; 1:1000, HRP-labeled goat anti-mouse IgG for GAPDH, Beyotime) for 1 h at room temperature. The protein bands were visualized with an ImageQuant LAS 4000 (Fujifilm, Tokyo, Japan) and quantified using ImageJ 1.50i software (National Institutes of Health).

### 4.10. Immunofluorescence

The frozen sections of subcutaneous fat stored at −20 °C until staining. The frozen sections prepared from the refrigerator were melted on a ventilated stage for 20 min. The frozen sections were fixed with 4% (*v*/*v*) paraformaldehyde for 1 h at 4 °C, washed three time with PBS, and then permeabilized with 0.2% (*v*/*v*) Triton X-100/PBS for 15 min. After treating with 3% (*w*/*v*) bovine serum albumin (BSA)/PBS for 30 min at room temperature, frozen sections were incubated overnight at 4 °C with primary rabbit anti-RUNX1T1 antibody (Proteintech) diluted 1:100 in 1% (*w*/*v*) BSA/PBS. After washing three times with PBS, the frozen sections were incubated with 594-conjugated donkey anti-rabbit antibody (1:200 dilution; Abcam, Cambridge, UK) in the dark for 2 h at room temperature. Subsequently, samples were stained with DAPI (Beyotime, Shanghai, China) for 10 min at room temperature. All samples were examined under a confocal laser scanning microscope (Zeiss LSM 710 META, Jena, Germany).

### 4.11. Statistical Analysis

All experiments were carried out in triplicate. All values were expressed as the mean ± SEM. Statistical analysis was performed using the SPSS software (version 24.0, SPSS Inc., Chicago, IL, USA) by either an independent Student’s *t*-test or one-way analysis of variance (ANOVA) with Tuckey *post hoc* analysis. For all analyses, *p* < 0.05 is indicated as a statistically significant.

## 5. Conclusions

In conclusion, this study showed that the expression of the long isoform of *RUNX1T1* was higher in the subcutaneous fat of Hu sheep. In addition, an increase in the long isoform of *RUNX1T1* was observed during ovine preadipocyte differentiation, strongly indicating that the long isoform of *RUNX1T1* may be involved in ovine adipogenesis. This is the first report that *RUNX1T1-L* knockdown promoted preadipocyte differentiation and lipid accumulation, indicating a negative relationship between *RUNX1T1-L* expression and ovine preadipocyte differentiation. However, little is known about the mechanism by which the long isoform of *RUNX1T1* regulates preadipocyte differentiation. Therefore, further studies are needed to elucidate the role of the long isoform of *RUNX1T1* in preadipocyte differentiation.

## Supplementary Materials

Supplementary materials can be found at http://www.mdpi.com/1422-0067/19/5/1300/s1.

## Abbreviations

ADIPOQ	Adiponectin
C/EBP	CCAAT/enhancer-binding protein
FTO	Fat mass and obesity-associated
GAPDH	Glyceraldehyde-3-phosphate dehydrogenase
LPL	Lipoprotein lipase
PPARγ	Peroxisome proliferator-activated receptor gamma
RUNX1T1	Runt-related transcription factor 1 translocation partner 1
