# Supplementary material for: Characterization of RUNX1T1, an Adipogenesis Regulator in Ovine Preadipocyte Differentiation
Source: Int J Mol Sci. 2018 Apr 26;19(5):1300. doi: 10.3390/ijms19051300 (PMC5983735; doi:10.3390/ijms19051300)
Supplement: Supplementary file 1 [file ijms-19-01300-s001.pdf]

Table S1. Details of primer sequences used for this study.

Items	Primer sequence (5′-3′)	Genebank No.	Fragment size (bp)	Target
GAPDH	F: CGACTTCAACAGCGACACTCAC<br>R: CCCTGTTGCTGTAGCCGAATTC	NM_001034034.1	119	GAPDH gene qRT-PCR
RUNX1T1-CDS	F: AGAGCGATTGGTGGAGAATGAT<br>R: CTAGCGAGGTGTCGTCTCT	XM_015097931.1	1829	RUNX1T1 gene full-length cDNA amplification
RUNX1T1-I	F: CAAACAGAACCCTGCCCAGT<br>R: TGTTTCCATTCCTCTGCCCA	XM_015097931.1	456	Identify RUNX1T1 isoform
RUNX1T1-II	F: GCGAACTCCAGACAGAACCA<br>R:CAAACGGTAATGCTGCGGTG	XM_015097931.1	178	RUNX1T1-L isoform Real-time PCR
FTO	F:AACGAGAGCGCGAAGCTAAG<br>R:CCGATGAGGATGCGAGAGAC	NM_001104931.1	274	FTO gene Real-time PCR
PPARγ	F:ACTTTGGGATCAGCTCCGTG<br>R: GTCAGCTCTTGGGAACGGAA	NM_001100921	159	PPARγ gene Real-time PCR
C/EBPα	F:ACAGCAACGAATACCGGGTG<br>R: TTCTGTTGCGTCTCCACGTT	NM_001308574.1	97	C/EBPα gene Real-time PCR
ADIPOQ	F:GGAGATCCAGGTCTCGTTGG<br>R:TTTCTGCCTGGGACTCCTGG	NM_001308565	98	ADIPOQ gene Real-time PCR
LPL	F:GACTCCAACGTCATCGTGGT<br>R:CCCCAAGGCTGTATCCCAAG	NM_001009394.1	172	LPL gene Real-time PCR

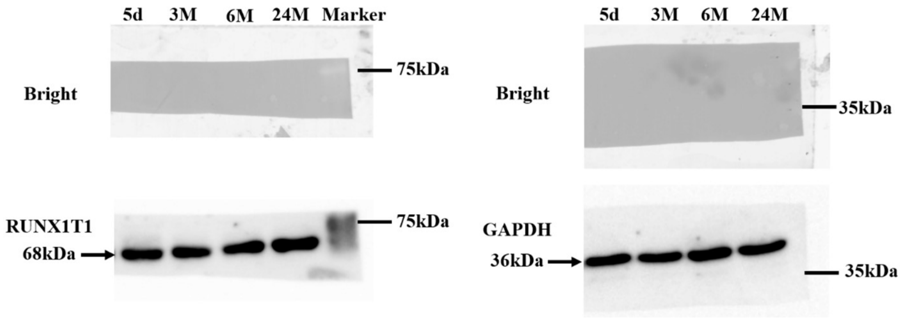

Figure S1. Original western blots image of RUNX1T1 and GAPDH from subcutaneous fat of Hu sheep.
